# The Chromatin Modifier Protein FfJMHY Plays an Important Role in Regulating the Rate of Mycelial Growth and Stipe Elongation in *Flammulina filiformis*

**DOI:** 10.3390/jof8050477

**Published:** 2022-05-03

**Authors:** Jian Li, Yanping Shao, Yayong Yang, Chang Xu, Zhuohan Jing, Hui Li, Baogui Xie, Yongxin Tao

**Affiliations:** 1 College of Horticulture, Fujian Agriculture and Forestry University, Fuzhou 350002, China; jianli0226@163.com (J.L.); huyx98@163.com (Y.S.); yangyy1839@163.com (Y.Y.); xc0406xn@163.com (C.X.); chenyz805236513@163.com (Z.J.); 2 Mycological Research Center, College of Life Sciences, Fujian Agriculture and Forestry University, Fuzhou 350002, China; mrcfafu@163.com; 3 Institute of Cash Crops, Hebei Academy of Agriculture and Forestry Sciences, Shijiazhuang 050051, China; lihuiviphappy@163.com

**Keywords:** *Flammulina filiformis*, stipe elongation, mycelial growth, chromatin modifier protein, cell wall composition

## Abstract

Stipe elongation is an important process in the development of the fruiting body and is associated with the commodity quality of agaric fungi. In this study, *F. filiformis* was used as a model agaric fungus to reveal the function of the chromatin modifier gene containing the JmjC domain in stipe elongation. First, we identified a JmjC domain family gene (*FfJmhy*) with a 3684 bp length open reading frame (ORF) in *F. filiformis*. *FfJmhy* was predicted to have a histone H3K9 demethylation function, and was specifically upregulated during stipe rapid elongation. Further investigation revealed that the silencing of *FfJmhy* inhibited the mycelial growth, while overexpression of this gene had no effect on the mycelial growth. Comparative analysis revealed that the stipe elongation rate in *FfJmhy* overexpression strains was significantly increased, while it was largely reduced when *FfJmhy* was silenced. Taken together, these results suggest that *FfJmhy* positively regulates the mycelial growth and controls the elongation speed and the length of the stipe. Moreover, cell wall-related enzymes genes, including three exo-β-1,3-glucanases, one β-1,6-glucan synthase, four chitinases, and two expansin proteins, were found to be regulated by *FfJmhy*. Based on the putative functions of *FfJmhy*, we propose that this gene enhances the transcription of cell wall-related enzymes genes by demethylating histone H3K9 sites to regulate remodeling of the cell wall in rapid stipe elongation. This study provides new insight into the mechanism of rapid stipe elongation, and it is important to regulate the commodity quality of agaric fungi.

## 1. Introduction

Edible fungi are nutritious and healthy food materials that mainly include agaric fungi (umbrella-shaped), polyporus fungi, and jelly fungi. At present, agaric fungi account for a large proportion of edible fungi that can be cultivated commercially. The fruiting body of agaric fungi is mainly composed of the pileus and stipe. The main edible part of some agaric fungi, such as *Lentinula edodes*, is the pileus, in which case a small and short stipe meets the requirements of high commodity value. In others, such as *Flammulina filiformis* (East Asian strain, a variety of European *Flammulina velutipes* [1]), the main edible part is the stipe. In this case, a long stipe provides high yields and great economic benefits. Therefore, it is important to study the factors affecting stipe elongation and its regulatory mechanism in agaric fungi to improve their commercial value and economic benefits.

Previous studies have found that the plot of the stipe elongation rate is not a straight line but a sigmoid curve [2]; that is, the rate is slow in the early stage, fast in the middle stage, and slow in the later stage. Accordingly, the development of *F. filiformis* in industrialized cultivation usually includes three stages: (1) the young fruiting body stage (from the 1st to the 4th day after primordia formation, when the average speed of stipe elongation is 0.68 cm/d), (2) the elongation stage (from the 5th to the 10th days, when the average speed is 1.78 cm/d), and (3) the maturation stage (from the 11th to the 12th days, when the average speed is 1.0 cm/d) [2]. Stipe elongation in other agaric fungi, such as *Coprinus lagopus* [3] and *Agaricus bisporus* [4], also follows a similar pattern. In *Coprinopsis cinerea*, the stipe extends from 20 mm to approximately 100 mm in less than 12 h during the elongation stage [5], while the stipe of *Volvariella volvacea* extends by 5–7 cm within 5–12 h in the rapid elongation stage (approximately 3–5-fold changes compared to the previous stage) [6]. The characteristic of rapid stipe elongation (the growth rate in the elongation stage is significantly higher than those in other stages) is also present in most agaric fungi [7].

Previous studies have found that the rapid elongation of the stipe mainly occurs through the rapid elongation of cells rather than a large increase in the number of cells [8,9]. This rapid elongation of cells is accompanied by remodeling of the cell wall structure, which is closely related to changes in the main components of the cell wall, β-glucan, and chitin [10]. It has been reported that during the stipe elongation process in *C. cinerea*, the glucanase I and glucanase II contents in the stipe significantly increased by 1.6 and 8.2 times, respectively, indicating that glucanase plays an important role in stipe elongation [9]. Glucanase can also cooperate with chitinase to regulate the expansion of the stipe cell wall by remodeling or inducing the expansion of the stipe cell wall [11]. Three exo-β-1,3-glucanase family genes *Ffexg1*, *Ffexg2*, and *Ffexg3* have been identified in *F. filiformis*. Among them, the transcription levels of *Ffexg2* and *Ffexg3* reach a maximum during the elongation stage [12]. It has also been confirmed that the *fv-gs6* gene (β-1,6-glucan synthase gene) has the highest expression levels during stipe elongation in *F. filiformis* [13]. The silencing of chitinase-encoding genes (*ChiE1* and *ChiIII*) resulted in stipe elongation phenotypic defects, inhibited stipe elongation-related growth, and decreased stipe cell wall elongation activity in *C. cinerea* [11]. In addition, fungal expansin-like proteins have been found to bind to chitin and promote its hydrolysis [14,15,16]. Therefore, glucanase, chitinase, and expansin-like proteins all participate in the rapid elongation of the stipe. However, the regulatory mechanisms of genes specifically involved in stipe elongation are currently seldom studied.

Genes that regulate rapid stipe elongation in *V. volvacea* have been identified through transcriptome sequencing, notably chitin-binding domain transcription factors [6,17], protein kinases [18], and Jumonji family genes. Jumonji family genes encode important chromatin modification proteins that function as histone demethylases, and are closely associated with gene transcription regulation, heterochromatin formation, genome integrity, and cell development [19,20]. DeJong et al. [21] observed that the growth rate of the mycelia of *Jmj3* knockdown mutants slowed down in *Schizophyllum commune*. In addition, five proteins containing JmjC domains (*Jhd1p*, *Jhd2p*, *Rph1p*, *Gis1p*, and *Ecm5p*) were identified in yeast, and they play important roles in the yeast life cycle [22]. In plants, Saze et al. [23] found that mutation of the JmjC family gene *Ibm1* caused plant dwarfing and leaf atrophy in *Arabidopsis*. These results suggest that Jumonji family genes are involved in growth and development. However, there is no report of Jumonji family genes in the development of the fungal fruiting body, and their detailed functions and regulatory mechanisms in fungi, especially agaric fungi, are still unclear.

*F. filiformis* is a typical agaric fungus and one of the most popular edible fungi with high nutritional and medicinal value [24]. It has the highest degree of industrial cultivation and can also be cultivated in the laboratory. Based on its stipe elongation characteristics, *F. filiformis* can be used as a model agaric fungus to reveal the regulatory mechanism of rapid stipe elongation. In this study, we determined the effect of *FfJmhy* silencing and overexpression in *F. filiformis*. We identified several genes whose transcription is affected by *FfJmhy*. The results will provide a better understanding of the regulatory network of stipe elongation, and be of great significance for radically regulating the stipe growth rate and length in mushroom production and improving the commodity quality of agaric fungi.

## 2. Materials and Methods

### 2.1. Strains and Culture Conditions

*F. filiformis* monokaryotic strains L11 and L22, as well as the dikaryotic strain FL19 (a hybrid obtained by mating L11 and L22), were obtained from the Fujian Edible Fungi Germplasm Resource Collection Center of China. They were maintained with periodic transfers on potato dextrose agar (PDA) at 25 °C. *Agrobacterium tumefaciens* AGL-1 was provided by Tiangen BioTech Co., Ltd. (Beijing, China).

Cultivation of the fruiting body of the FL19 strain, as well as all transformant strains, was performed per the method described by Tao et al. [25] with some modifications. The cultivation medium was composed of 49% sawdust, 30% cottonseed shells, 20% wheat bran, and 1% pulverized lime with 60% water saturation and packed in cultivation bottles (200 g per bottle). All transformants and the WT with an equal number of equipotent PDA blocks were inoculated and cultivated at a constant 25 °C. When the cultivation medium was completely occupied by mycelia, it was placed in an environment of 8~12 °C and 85~90% humidity to induce the formation of primordia and the development of the fruiting body.

### 2.2. Gene Sequence and Structure Analysis of FfJmhy in F. filiformis

According to the Jumonji family gene (GME9838_g) with specifically high transcription in the stipe elongation stage of *V. volvacea* screened by Tao et al. [6], its orthologous gene *FfJmhy* in the genome of *F. filiformis* L11 (NCBI accession No. PRJNA191865) was identified by local BLASTp searching. The sequence of *FfJmhy* was then retrieved from the genome of the *F. filiformis* L11 strain, and their orthologous relationship was verified by reciprocal BLAST. The predicted gene model was confirmed by sequencing of the full-length open reading frame (ORF) obtained by PCR with primers FfJmhy-oeF and FfJmhy-oeR (Supplementary Material Table S1).

### 2.3. Total DNA and RNA Extraction and RT-qPCR

All the samples used for DNA and RNA extraction were collected with equal mass (0.5 g). Total DNA was extracted from the mycelia of *F. filiformis* using modified CTAB [26]. Total RNA was extracted according to the standard method described in the instructions of the Omega E. Z.N.A. Plant RNA Kit, in which the treatment with DNase was performed (Omega Bio-Tek, Norcross, GA, USA).

Extracted RNA was quantified using a NanoND-1000 spectrophotometer (NanoDrop Technologies, Wilmington, DE, USA). Only RNA samples with A260/A280 ratios between 1.9 and 2.1 and A260/A230 ratios greater than 2.0 were used for cDNA synthesis. The concentration of extracted total RNA was diluted to 500 ng/μL for each sample. The cDNA was synthesized using 1000 ng of RNA for each sample, according to the instructions of a TransScript All-in-One First-Strand cDNA Synthesis SuperMix for qPCR kit, in which the treatment with gDNA remover was performed (Transgen, Beijing, China).

Real-Time quantitative PCR (RT-qPCR) was performed using a CFX96 Real-Time PCR Detection System (Bio–Rad, Hercules, CA, USA). RT-qPCR amplification included a denaturation step of 10 s at 95 °C, followed by 40 cycles of 5 s at 95 °C and 30 s at primer-specific annealing temperatures, according to the instructions of PerfectStart Green qPCR SuperMix (Transgen, Beijing, China). Six candidate internal control genes including *SPRYp*, *L-asp*, *Vps26*, *ACTB*, *Ras,* and *GAPDH* were first selected according to the results identified from the *F. filiformis* RNA-Seq data analysis by Tao et al. [27], and their stability was further evaluated among the WT and transformants using geNorm software [28]. According to the stability ranking results of six candidates and geNorm’s recommendations (Supplementary Material Figure S1), *ACTB*, *Ras,* and *GAPDH* were used as final internal control genes for the normalization of RT-qPCR in this study. All the primers for RT-qPCR designed in exon regions are shown in Table S1 (Supplementary Material). In addition, the amplification efficiency of all the primers for RT-qPCR was detected, to ensure that they were between 90 and 110%. The R^2^ of standard curves were greater than 0.98, and the standard deviation of C(t) values of three technical replications was less than 0.2, as required by Bustin et al. [29] in the MIQE. The above processes and standardized controls were performed strictly to ensure the RT-qPCR was MIQE-compliant, recommended by Bustin et al. [29,30]. The relative gene expression levels were determined according to the 2^−ΔΔCt^ method [31].

### 2.4. Bioinformatics Analysis of the FfJMHY

The ExPASy Protparam (http://www.expasy.ch/tools/protparam.html (accessed on 1 December 2021)) [32] was used to analyze the basic physical and chemical properties of FfJMHY. SMART (https://smart.embl.de (accessed on 1 December 2021)) [33] and cNLS Mapper (https://nls-mapper.iab.keio.ac.jp/cgi-bin/NLS_Mapper_form.cgi (accessed on 1 December 2021)) [34] were used to predict the conserved domain and the nuclear localization signal (NLS), respectively. The phylogenetic tree was constructed using ClustalW 1.83 [35] and MEGA 7.0 [36] by the Neighbor-joining method and a bootstrap of 1000 replications. The Softberry website (http://linux1.softberry.com (accessed on 4 December 2021)) [37] was used to predict the subcellular localization in silico. PlantCare (https://bioinformatics.psb.ugent.be/webtools/plantcare/html (accessed on 4 December 2021)) [38] was used to predict the possible cis-acting elements in the promoter region of the *FfJmhy* gene.

### 2.5. FfJmhy Overexpression and Silencing Plasmid Construction

**Figure 1:**
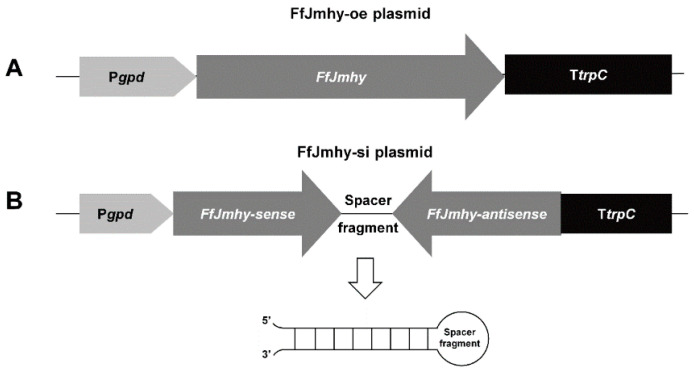
Construction strategy of *FfJmhy* overexpression and silencing plasmids. The *gpd* promoter and *trpC* terminator are represented by a light gray arrow and black rectangle, respectively. (**A**) Construction strategy of *FfJmhy* overexpression plasmid. The dark gray arrow represents the ORF sequence of *FfJmhy*. (**B**) Construction strategy of *FfJmhy* silencing plasmid. The dark gray arrow represents the *FfJmhy*-sence and antisense fragments, which are reverse complementary and can form hairpins. The detailed construction methods are shown in Section 2.5.

The pBHg-BCA1 [39] binary vector was used to construct the *FfJmhy* overexpression and silencing plasmids with the hygromycin B phosphotransferase gene (*Hpt*) for transformant verification. The ORF of *FfJmhy* was inserted into pBHg-BCA1 to obtain the overexpression plasmid (FfJmhy-oe, Figure 1A). To construct the *FfJmhy* silencing plasmid (FfJmhy-si), the primers FfJmhy-sense-F and FfJmhy-sense-R were used to amplify the forward sequence of *FfJmhy* and were inserted into pBHg-BCA1 to form the FfJmhy-si precursor. Next, the primers FfJmhy-antisense-F and FfJmhy-antisense-R were used to amplify the reverse sequence and spacer of *FfJmhy*, which were connected to the FfJmhy-si precursor to form the final FfJmhy-si plasmid (Figure 1B). All primers used are listed in Table S1 (Supplementary Material).

### 2.6. FfJmhy Overexpression and Silencing Strain Construction

The obtained FfJmhy-oe and FfJmhy-si plasmids were transferred into the FL19 strain by *A. tumefaciens* infection according to a previously reported protocol [40,41]. The transformant candidates were cultured on a complete yeast medium (CYM: 1% maltose, 2% glucose, 0.2% yeast extract, 0.2% tryptone, 0.05% MgSO_4_·7H_2_O, 0.46% KH_2_PO_4_, pH = 5.5) containing hygromycin B (30 μg/mL) for five consecutive generations and then confirmed by *Hpt* gene PCR using the primers Hpt-F and Hpt-R (Table S1). Total RNA from the positive transformants was used for the detection of *FfJmhy* expression levels by RT-qPCR.

A control strain was obtained by *A. tumefaciens* with an empty vector (pBHg-BCA1) that did not contain the target gene, and it was confirmed by genome sequencing that the resistance gene fragment from the empty vector was inserted into an intergenic region, meaning that genes were not damaged in the control strain. The growth phenotype of the control strain was consistent with the wild type (WT).

### 2.7. Phenotypic Analysis of the Transformants

The transformed strains of *FfJmhy* and the wild type were inoculated on the CYM medium and incubated at 25 °C in darkness. The starting and ending points of mycelial growth were marked, and the growth rate was calculated on the 6th day after incubation.

First, the mycelial growth was recorded from starting and ending positions in the cultivation bottle, and then the growth rate was calculated. When the mycelia of all strains were overgrown, both scratching and inducing primordia were incubated at 8~12 °C. The average numbers of fruiting bodies of all tested strains were counted according to the method of Tao et al. [25]. The differential phenotypes of stipe elongation in all tested strains were photographed and measured in both the elongation stage (the defining feature is that the pileus is fully formed but not expanded) and the maturation stage (the defining feature is that the pileus is completely horizontal, and the spores are scattered). Stipes randomly selected and marked in the young stage in the different cultivation bottles were continuously tracked, and their lengths were measured daily until the end of the maturation stage.

### 2.8. Statistical Analysis

To ensure that the observed trends were reproducible, all experimental data were obtained in three independent biological replicates. The values in the figures are the means ± standard deviation (SD) of three independent experiments. Student’s *t* test was used to analyze the significance between two samples, and one-way ANOVA was used to analyze the significance of multiple comparisons. The different statistical methods and tests are specified in the corresponding figure legend.

## 3. Results

### 3.1. Gene Structure, Protein Structure, and Phylogeny of FfJmhy

**Figure 2:**
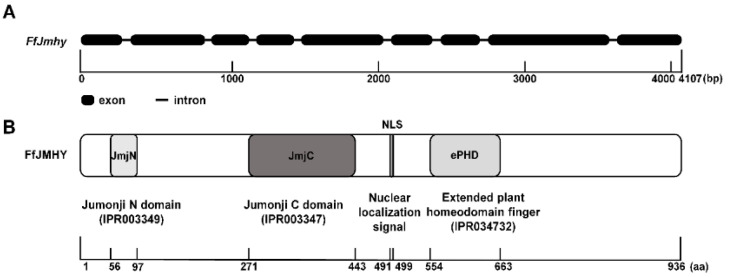
Gene and protein structures of *FfJmhy* sequence in *F. filiformis*. (**A**) Structure of *FfJmhy* gene in *F. filiformis*. Thick lines represent exons and thin lines represent introns. (**B**) Structure and conserved domains of FfJMHY protein in *F. filiformis*. NLS: predicted nuclear localization signal.

The gDNA sequence of *FfJmhy* is 4107 bp in length, composed of 9 exons and 8 introns, and contains a 3684 bp ORF (Figure 2A) (GenBank accession No. MG670543). ProtParam analyzed its physical and chemical properties, and the results showed that the *FfJmhy* gene encodes a protein of 936 amino acids (aa) with a predicted molecular mass of 102.8 KDa and an isoelectric point of 8.75. As shown in Figure 2B, SMART analysis of the domains showed that FfJMHY contains three conserved domains. The JmjN domain (Jumonji N domain, IPR003349) and JmjC domain (Jumonji C domain, IPR003347) are typical histone demethylases (JmjC family), indicating that FfJMHY may have histone demethylase activity. The ePHD domain (extended plant homeodomain, IPR034732) can specifically recognize the methylation (modification) sites of histones. The function of the conserved domain indicates that the protein is a member of Jumonji domain-containing protein 2 (Figure 2B), named FfJMHY (GenBank accession no. AUT12060) in *F. filiformis*.

**Figure 3:**
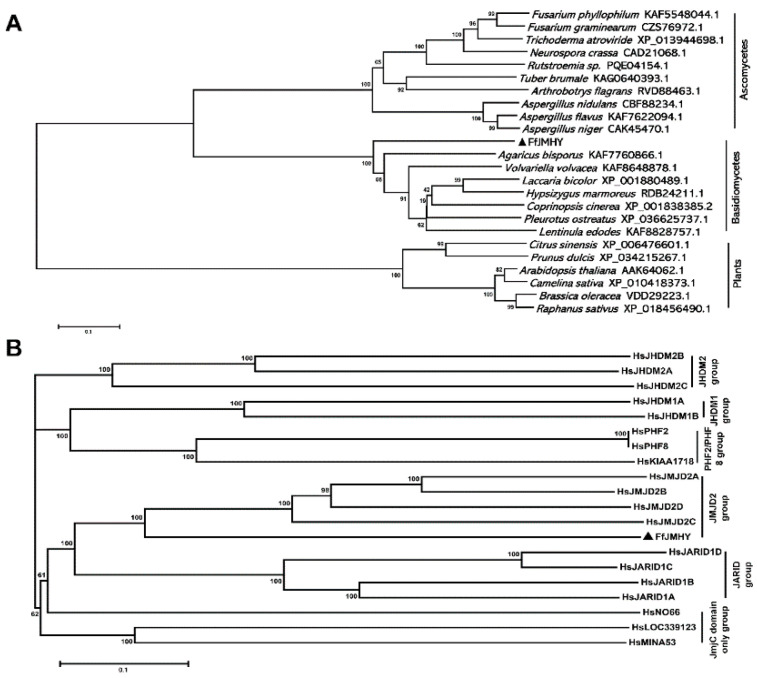
Phylogeny and subfamily location analysis of FfJMHY. Black triangle represents FfJMHY in *F. filiformis*. (**A**) Phylogenetic tree of FfJMHY and JmjC proteins from other species including plants, basidiomycetes, and ascomycetes. (**B**) Subfamily location analysis of FfJMHY. The six evolutionarily conserved subfamilies of JmjC domain proteins in *Homo sapiens* referred by Klose et al. [44] are indicated on the right of the tree.

Furthermore, the results of phylogenetic tree analysis showed that FfJMHY and the JMHYs of *A. bisporus*, *V. volvacea*, *Laccaria bicolor*, *Hypsizygus marmoreus*, *C. cinerea*, *Pleurotus ostreatus*, and *L. edodes* belong to the same basidiomycete branch. The JMHYs of *Fusarium phyllophilum*, *Trichoderma atroviride*, *Neurospora crassa*, *Rutstroemia* sp., *Tuber brumale*, *Arthrobotrys flagrans*, *Aspergillus nidulans*, *Aspergillus flavus*, and *Aspergillus niger* belong to the ascomycete branch, indicating that JMHY evolution was consistent with biological evolution (Figure 3A). In addition, the JmjC family is composed of multiple subfamilies, such as JHDM2, JARID, JmjC domain only, JMJD2, and JHDM1. The phylogenetic tree was constructed by combining FfJMHY with proteins known to contain the JmjC domain in humans. The results showed that FfJMHY belongs to the JMJD2 branch (Figure 3B). This subfamily has histone H3K9 and H3K36 demethylation functions [42,43], suggesting that FfJMHY might have a similar function.

### 3.2. Bioinformatics Analysis of FfJMHY

Based on cNLS Mapper analysis, we also found a nuclear localization signal in FfJMHY located at 491–499 aa (Figure 2B). The subcellular localization prediction in silico also showed that FfJMHY is located in the nucleus, suggesting that FfJMHY is a chromatin modifier protein that can enter the nucleus to perform its functions.

The PlantCARE prediction results showed that the *FfJmhy* promoter region contains cis-response elements related to environmental factors such as low temperature, drought, and light and has hormone response elements such as methyl jasmonate and auxin, indicating that the transcription of *FfJmhy* may be involved in the development of the fruiting body in mushrooms (Supplementary Material Table S2 and Figure S2).

### 3.3. FfJmhy Is Significantly Highly Expressed during the Rapid Elongation of the Stipe

**Figure 4:**
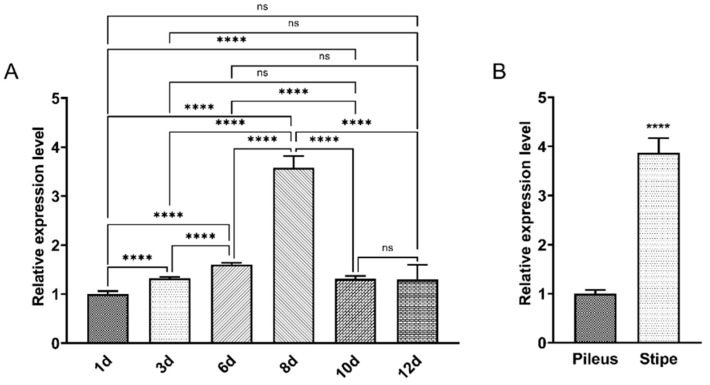
Expression levels of *FfJmhy* in different developmental stages and tissue. (**A**) The expression levels of *FfJmhy* in the fruiting body of the strain FL19 on the 1st, 3rd, 6th, 8th, 10th, and 12th days of primordia appearance. The values are the means ± SD of three independent experiments. Asterisks indicate significant differences (Dunnett T3’s multiple comparisons test: **** *p* < 0.0001, ns: not significant). (**B**) Expression levels of *FfJmhy* in the pileus and stipe of the strain FL19 fruiting body on 8th day after primordia appearance. The values are the means ± SD of three independent experiments. Asterisks indicate significant differences compared to pileus (Student’s *t* test: **** *p* < 0.0001).

To explore its effect on the growth and development of *F. filiformis*, we performed RT-qPCR analysis of the *FfJmhy* gene transcription at different developmental stages in *F. filiformis*. The expression levels of *FfJmhy* showed a trend of first increasing and then decreasing throughout the development of the stipe. The highest expression levels were shown on the 8th day of stipe development, reaching 3.5 times that on the first day, and it was significantly higher than those in other stages (Figure 4A). Fruiting body development in *F. filiformis* lasted for approximately 12 days from the primordia to maturation stage under industrialized cultivation conditions. The 5th to the 10th days comprised the fastest stipe elongation stage (named the elongation stage) [2]. The expression levels of the *FfJmhy* gene were consistent with the pattern of stipe elongation, and transcription reached the highest levels in the rapid stipe elongation stage, indicating that *FfJmhy* may be involved in the regulation of rapid stipe elongation in *F. filiformis*. We also performed RT-qPCR of the stipe and pileus in the elongation stage. The results showed that the expression levels of *FfJmhy* in the stipe upregulated 3.8 times compared to that in the pileus (Figure 4B), suggesting that *FfJmhy* may be specifically expressed in the stipe.

### 3.4. Obtaining FfJmhy Overexpression and Silencing Mutants

**Figure 5:**
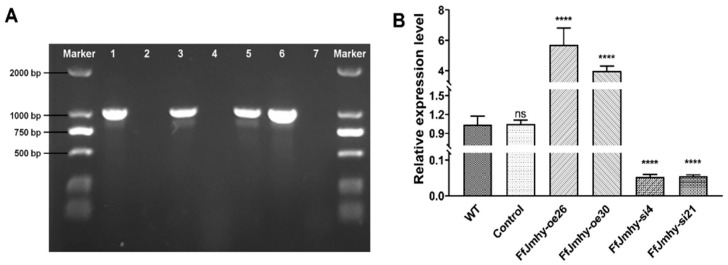
Validation of *FfJmhy* transformants. (**A**) PCR verification results of *Hpt* gene in the transformants. Numbers 1–7 represent the transformants of FfJmhy-oe26, FfJmhy-oe29, FfJmhy-oe30, FfJmhy-si1, FfJmhy-si4, FfJmhy-si21, and WT, respectively. The 2000 bp markers are shown on both sides of the electropherogram. (**B**) Verification of the expression levels of *FfJmhy* gene in the transformants. Details of the control strain are described in the Section 2.6. The values are the means ± SD of three independent experiments. Asterisks indicate significant differences compared to WT (FL19) (Dunnett T3’s multiple comparisons test: **** *p* < 0.0001, ns: no significant).

To verify the regulatory roles of *FfJmhy* in stipe elongation in *F. filiformis*, we constructed *FfJmhy* overexpression and silencing transformant strains. First, six transformant candidates (FfJmhy-oe26, FfJmhy-oe29, FfJmhy-oe30, FfJmhy-si1, FfJmhy-si4, and FfJmhy-si21) were screened according to hygromycin resistance. Two false-positive strains (FfJmhy-oe29 and FfJmhy-si1) were excluded by PCR verification using primers Hpt-F and Hpt-R, and the four remaining transformant strains (FfJmhy-oe26, FfJmhy-oe30, FfJmhy-si4, and FfJmhy-si21) were confirmed as positive mutants and used for further RT-qPCR analysis (Figure 5A). The expression levels of *FfJmhy* in FfJmhy-oe26 and FfJmhy-oe30 were increased by 5.8 times and 3.9 times, respectively, compared with that in the WT. Moreover, the expression levels of *FfJmhy* in FfJmhy-si4 and FfJmhy-si21 were reduced by 94.8% and 94.5%, respectively (Figure 5B).

### 3.5. FfJmhy Silencing Reduces the Normal Growth of Mycelia

**Figure 6:**
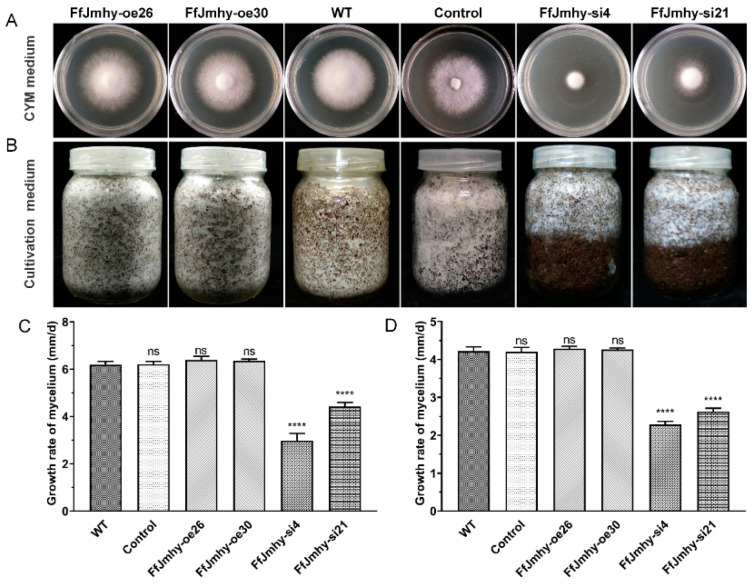
Vegetative growth phenotype of *FfJmhy* transformants. Details of the control strain are described in Section 2.6. (**A**) Mycelial phenotype of *FfJmhy* transformants on the 6th day growth on CYM medium. (**B**) Mycelial phenotype of *FfJmhy* transformants growth on the 25th day in the cultivation medium described in Section 2.1. (**C**) Growth rate of mycelia of *FfJmhy* transformants on CYM medium. (Dunnett T3’s multiple comparisons test: **** *p* < 0.0001, ns: not significant). (**D**) Growth rate of mycelia of *FfJmhy* transformants in the cultivation medium. (Tukey’s multiple comparisons test: **** *p* < 0.0001, ns: not significant). In (**C**,**D**), the values are the means ± SD of three independent experiments. Asterisks indicate significant differences compared to WT (FL19).

By observing the morphology of the mycelia on CYM medium, we found that the growth rate of the mycelia in the silencing strains was significantly lower than that in the WT. The mycelial growth rates of the FfJmhy-si4 and FfJmhy-si21 strains were significantly reduced to 2.98 mm/d and 4.49 mm/d and were 51.9% and 27.6% lower than those in the WT, respectively (6.2 mm/d) (Figure 6A,C). However, the growth rates of the overexpression strains FfJmhy-oe26 and FfJmhy-oe30 (6.39 mm/d and 6.34 mm/d, respectively) were similar to those of the WT.

We studied the effect of *FfJmhy* overexpression and silencing on vegetative growth in the cultivation medium. The growth rates of mycelia in FfJmhy-si4 and FfJmhy-si21 were only 2.28 mm/d and 2.62 mm/d, which were lower than those in the WT (4.21 mm/d) by 45.9% and 37.9%, respectively (Figure 6B,D). This result suggested that *FfJmhy* overexpression (FfJmhy-oe26: 4.29 mm/d; FfJmhy-oe30: 4.27 mm/d) did not affect the growth of mycelia, while *FfJmhy* silencing largely inhibited mycelial growth.

### 3.6. FfJmhy Positively Regulates Stipe Development

**Figure 7:**
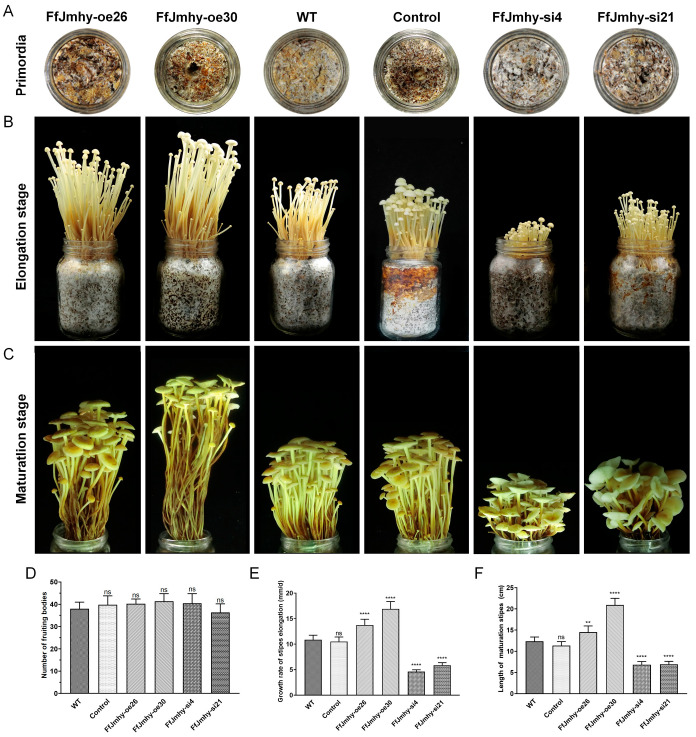
The phenotype of fruiting body of *FfJmhy* transformants. Details of the control strain are described in Section 2.6. (**A**) The phenotype of primordia formation of *FfJmhy* transformants on the 6th day after the stimulation. (**B**) The phenotype of the fruiting body of *FfJmhy* transformants in elongation stage. (**C**) The phenotype of the fruiting body of *FfJmhy* transformants in maturation stage. (**D**) The average number of the fruiting body of *FfJmhy* transformants (Tukey’s multiple comparisons test, ns: not significant). (**E**) The average speed of the stipe elongation of *FfJmhy* transformants in elongation stage (Dunnett T3’s multiple comparisons test: **** *p* < 0.0001, ns: not significant). (**F**) The average length of the stipe of *FfJmhy* transformants in maturation stage (Tukey’s multiple comparisons test: ** *p* < 0.01, **** *p* < 0.0001, ns: no significant). In (**D**–**F**), the values are the means ± SD of three independent experiments. Asterisks indicate significant differences compared to WT (FL19).

Primordia differentiation appeared in the overexpression and WT strains after 6 days of primordia induction. Primordia differentiation appeared in the silencing strain 2 days later (8 days after primordia induction) than in the WT and overexpression strains (Figure 7A). However, delayed primordia differentiation did not affect the formation of the fruiting body. Approximately 36 to 43 mature fruiting bodies were produced by the overexpression and silencing strains, with no significant difference from the WT (in which approximately 38 mature fruiting bodies were produced) (Figure 7D). This result suggested that the silencing and overexpression of *FfJmhy* did not affect the number of the fruiting body.

Pileus expansion in the overexpression and WT strains reached the maximum (maturation standard) on the 12th day, and the total fruiting body development cycle lasted 12 days (the elongation stage was from the 5th to the 10th day). In the two silencing strains, the pileus was not fully expanded on 12th day and expanded horizontally (maturation standard) on 14th day. Thus, the total fruiting body development cycle in *FfJmhy* silencing strains lasted 14 days (the elongation stage was from the 6th to the 12th day).

Compared with the WT, the rate of stipe elongation in the overexpression strains was significantly increased. The average stipe elongation rate of the WT was 10.8 mm/d. The stipe elongation rates of the FfJmhy-oe26 and FfJmhy-oe30 strains were 13.8 mm/d and 16.9 mm/d, respectively, which were 1.26 times and 1.55 times higher than that of the WT. In contrast, the stipe elongation rates of the FfJmhy-si4 and FfJmhy-si21 strains were 4.54 mm/d and 5.89 mm/d, respectively, which were reduced by 58.3% and 45.9% compared with the WT (Figure 7B,E). When all strains reached the maturation standard, the mature stipe lengths of FfJmhy-si4 and FfJmhy-si21 were 6.8 cm and 6.9 cm, respectively, while the mature stipe lengths of FfJmhy-oe26 and FfJmhy-oe30 were 14.6 cm and 20.8 cm, respectively (Figure 7C,F).

In three independent repeated experiments, it was consistently observed that the stipe elongation speed and the length of the stipe increased in the overexpression strain. The stipe elongation rate of the silencing strain was reduced, and the length of the stipe was shortened. These results indicate that *FfJmhy* plays a positive regulatory role in stipe elongation.

### 3.7. FfJmhy Regulates the Transcription of Glucanase and Glucan Synthase Genes

**Figure 8:**
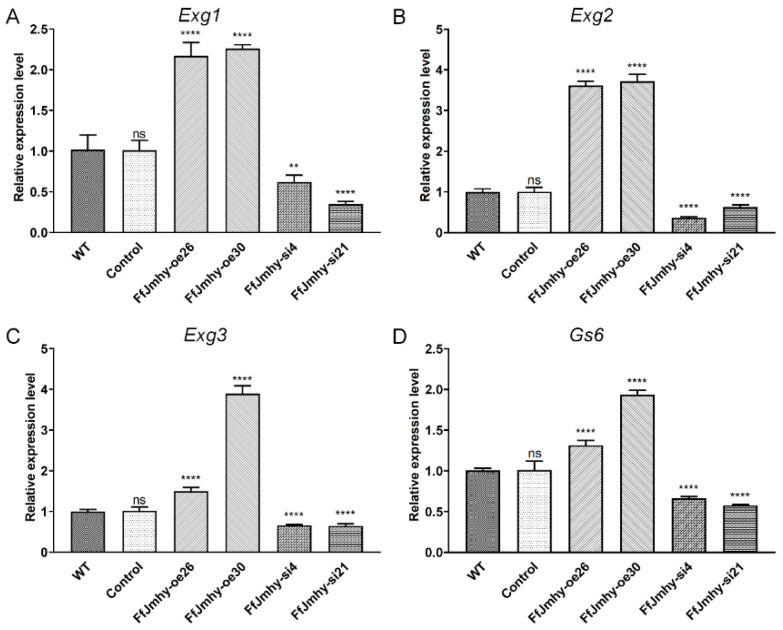
Relative expression levels of glucanase and glucan synthase-encoding genes in *FfJmhy* transformants in stipe elongation stage. Details of the control strain are described in Section 2.6. (**A**) Relative expression levels of glucanase *FfExg1* in *FfJmhy* transformants in stipe elongation stage (Dunnett T3’s multiple comparisons test: ** *p* < 0.01, **** *p* < 0.0001, ns: not significant). (**B**) Relative expression levels of glucanase *FfExg2* in *FfJmhy* transformants in stipe elongation stage (Dunnett T3’s multiple comparisons test: **** *p* < 0.0001, ns: not significant). (**C**) Relative expression levels of glucanase *FfExg3* in *FfJmhy* transformants in stipe elongation stage (Dunnett T3’s multiple comparisons test: **** *p* < 0.0001, ns: not significant). (**D**) Relative expression levels of glucan synthase *Gs6* in *FfJmhy* transformants in stipe elongation stage (Dunnett T3’s multiple comparisons test: **** *p* < 0.0001, ns: no significant). In (**A**–**D**), the values are the means ± SD of three independent experiments. Asterisks indicate significant differences compared to WT (FL19).

To further reveal the stipe elongation regulatory mechanism of *FfJmhy*, we investigated the transcription of glucanase and glucan synthase genes in overexpression and silencing strains. According to previous reports, there are three exo-β-1,3-glucanase (EXG, glycoside hydrolase family 55)-encoding genes in *F. filiformis*, namely, *Exg1*, *Exg2*, and *Exg3* [12]. We found that the expression levels of *Exg1* and *Exg2* in FfJmhy-oe26 were increased by 2.1 times and 3.6 times compared with those in the WT, respectively. The expression levels of *Exg1* and *Exg2* in FfJmhy-oe30 were increased by 2.3 times and 3.7 times compared with those in the WT, respectively. However, the relative expression levels of *Exg1* and *Exg2* in the FfJmhy-si4 strain decreased by 37.3% and 64.1%, respectively, and the relative expression levels of *Exg1* and *Exg2* in the FfJmhy-si21 strain decreased by 65.6% and 36.5%, respectively, compared with the WT (Figure 8A,B). In the FfJmhy-oe26 and FfJmhy-oe30 strains, the expression levels of *Exg3* were 1.5 times and 3.8 times that in the WT, respectively, while its expression levels were decreased by 33.9% and 35.3% in the FfJmhy-si4 and FfJmhy-si21 strains, respectively (Figure 8C).

According to the results of Long et al. [13], the β-1,6-glucan synthase (GS, glycoside hydrolase family 17)-encoding gene *Gs6* was upregulated in the elongation stage in *F. filiformis*. The RT-qPCR results showed that the expression levels of *Gs6* in the overexpression strains FfJmhy-oe26 and FfJmhy-oe30 were 1.3 times and 1.9 times higher than that in the WT, and the expression levels in FfJmhy-si4 and FfJmhy-si21 were reduced by 34.0% and 42.3%, respectively (Figure 8D).

### 3.8. FfJmhy Positively Regulates the Transcription of the Chitinase Gene

**Figure 9:**
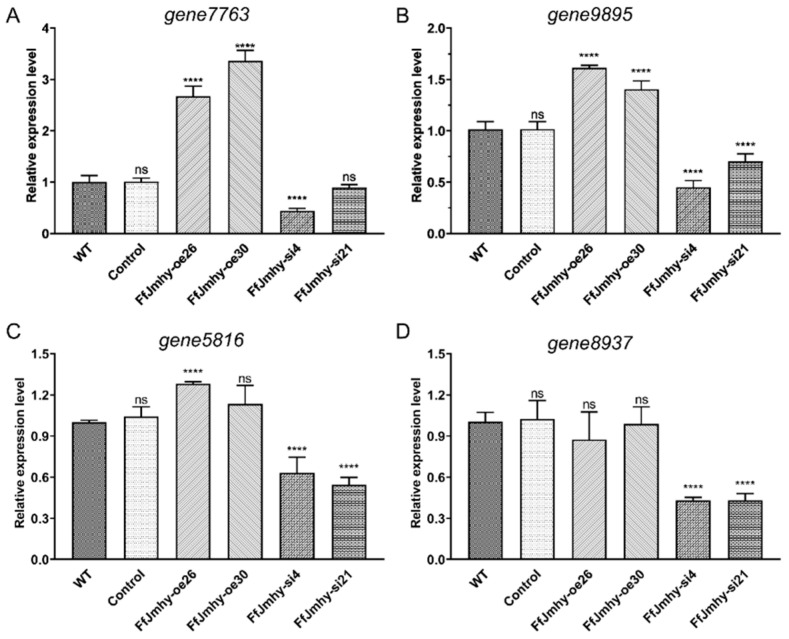
Relative expression levels of chitinase-encoding genes in *FfJmhy* transformants in stipe elongation stage. Details of the control strain are described in Section 2.6. (**A**) Relative expression levels of chitinase *gene7763* in *FfJmhy* transformants in stipe elongation stage (Dunnett T3’s multiple comparisons test: **** *p* < 0.0001, ns: not significant). (**B**) Relative expression levels of chitinase *gene9895* in *FfJmhy* transformants in stipe elongation stage (Tukey’s multiple comparisons test: **** *p* < 0.0001, ns: not significant). (**C**) Relative expression levels of chitinase *gene5816* in *FfJmhy* transformants in stipe elongation stage (Dunnett T3’s multiple comparisons test: **** *p* < 0.0001, ns: not significant). (**D**) Relative expression levels of chitinase *gene8937* in *FfJmhy* transformants in stipe elongation stage (Dunnett T3’s multiple comparisons test: **** *p* < 0.0001, ns: not significant). In (**A**–**D**), the values are the means ± SD of three independent experiments. Asterisks indicate significant differences compared to WT (FL19).

According to the RNA-Seq results of different developmental stages in *F. filiformis*, there are five chitinase (glycoside hydrolase family 18)-encoding genes highly expressed in the fruiting body stages [45], including *gene7763*, *gene2644*, *gene8937*, *gene9895,* and *gene5816*. The RT-qPCR results showed that four (*gene7763*, *gene8937*, *gene9895,* and *gene5816*) of these five chitinase genes, excluding *gene2644*, exhibited differential transcription in the *FfJmhy* transformant strains compared with the WT. In the FfJmhy-oe26 and FfJmhy-oe30 strains, *gene7763* increased by an average of 3.05 times, *gene9895* increased by an average of 1.5 times, and *gene5816* increased by an average of 1.15 times, while *gene8937* was not significantly changed. The expression levels in the FfJmhy-si4 and FfJmhy-si21 strains were as follows: *gene7763* was decreased by an average of 38%, *gene9895* was decreased by an average of 42.1%, *gene5816* was decreased by an average of 41.6%, and *gene8937* was decreased by an average of 57% (Figure 9).

### 3.9. FfJmhy Regulates the Transcription of Expansin Protein-Related Genes

**Figure 10:**
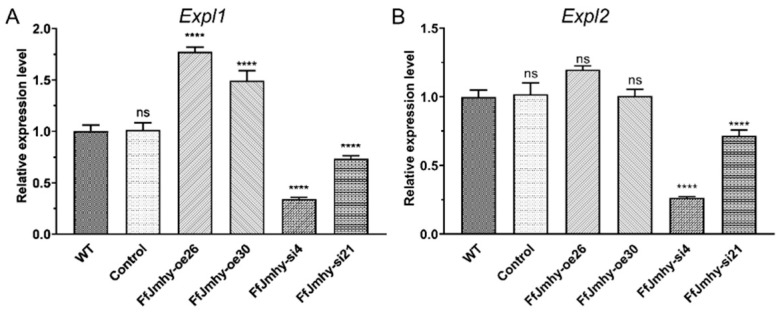
Relative expression levels of expansin-like genes in *FfJmhy* transformants in stipe elongation stage. Details of the control strain are described in Section 2.6. (**A**) Relative expression levels of expansin-like gene *Expl1* in *FfJmhy* transformants in stipe elongation stage (Dunnett T3’s multiple comparisons test: **** *p* < 0.0001, ns: not significant). (**B**) Relative expression levels of expansin-like gene *Expl2* in *FfJmhy* transformants in stipe elongation stage (Dunnett T3’s multiple comparisons test: **** *p* < 0.0001, ns: not significant). There is a confidence interval (CI) overlap between WT and FfJmhy-oe26, no significant difference between the two samples is acceptable. In A and B, the values are the means ± SD of three independent experiments. Asterisks indicate significant differences compared to WT (FL19).

Two expansin-like proteins, *Expl1* and *Expl2*, have been identified in *F. filiformis* and are upregulated during the elongation stage [15,46]. The RT-qPCR results showed that the expression levels of *Expl1* in FfJmhy-oe26 and FfJmhy-oe30 were 1.7 times and 1.5 times higher than that in the WT, respectively, and the expression levels in FfJmhy-si4 and FfJmhy-si21 were 65.9% and 23.3% lower than that in the WT, respectively. The expression levels of *Expl2* in FfJmhy-oe26 were increased by 1.2 times, while the expression levels in strains FfJmhy-si4 and FfJmhy-si21 were decreased by 73.1% and 30.1%, respectively, compared with the WT (Figure 10).

## 4. Discussion

Stipe elongation is the most important characteristic of fruiting body development in agaric fungi, and it is also significantly associated with the commodity quality and economic benefit of agaric mushrooms. Stipe elongation is affected by many environmental factors, but it is fundamentally controlled by intracellular regulatory factors. In a recent study on *V. volvacea*, cells in the stipe elongation stage extend rapidly rather than proliferate largely [6]. When a stipe cell elongates, the cell wall relaxes to expand its surface area and adapt to the enlarged protoplast [8,47]. This whole process requires precise and global regulation. Based on transcriptome screening and RT-qPCR verification, *Jmhy* was found to be specifically highly expressed during rapid stipe elongation in *F. filiformis*, suggesting that *FfJmhy* may be an overall and specific regulator of stipe rapid elongation. This function was confirmed by the silencing and overexpression of *FfJmhy*. The stipe elongation rate was reduced by 45.9% to 58.3% when *FfJmhy* expression levels were silenced by 94%, resulting in a reduction in the total stipe length by approximately 44.3%, and the opposite was observed with *FfJmhy* overexpression, although with different magnitudes of elongation.

According to clustering and structural analysis, FfJMHY belongs to the JMJD2 subfamily in the histone demethylase family (JmjC family), which has been reported to have histone H3K9- and H3K36-specific demethylation functions [37,38,48,49]. The methylation and demethylation of histones are epigenetic modification methods that are involved in the regulation of gene transcription. It has been proven that lysine methylation at the H3K9, H3K27, H3K79, and H4K20 sites exerts transcriptional repression [50,51,52]. Previous studies have shown that the H3K9 methylation site is mainly located in satellite repeats of chromosomes [53,54]. More than four satellite repeat regions existed in the promoters of *Exg2*, *Exg3*, *gene7763*, *gene5816,* and *Expl2* (Table S3), indicating the possibility that FfJMHY acts on these loci. Research in rice showed that increasing the transcription of *Jmj706* in the same JMJD2 subfamily enhanced the ability to demethylate histone H3K9 and reduced transcriptional inhibition caused by H3K9 methylation [55]. These results together with the current findings are evidence that the transcription levels of these genes maintained by consistent changes in the *FfJmhy* silencing and overexpression transformants give reason to believe that *FfJmhy* may enhance the transcription of these genes by demethylating histone H3K9 sites surrounded by these genes.

*FfJmhy* regulates β-1,3-glucan and chitin synthesis and decomposition enzyme genes, including *Exg2*, *Exg3*, *gene7763*, and *gene5816*, as well as expansin-like protein-encoding genes, such as *Expl2*, which has been proven to be involved in cell elongation by interacting with the cell wall. β-1,3-glucan and chitin form a scaffold structure and cross-linked with other polysaccharides and/or glycoproteins in the fungal cell wall [4,7,56,57,58]. Chitinase can destroy the cross-linking of matrix polysaccharides between the parallel chitin microfibril structures, such that the cell wall becomes partially parallel to the chitin microfibril. Structural breakage causes relaxation of the chitin microfibril structure, which facilitates the elongation of the cell wall. Exo-β-1,3-glucanase can cleave β-1,3-glucan side chains and the β-1,6-branched β-1,3-glucan backbone connected to chitin in the cell wall, loosening the cell wall structure and making it easier to extend. Upregulation of the transcription of chitinase and β-1,3-glucanase and their function of extending the cell wall in stipe elongation have been proven in *F. filiformis* as well as other agaric fungi, including *C. cinerea* and *V. volvacea* [11,17]. When the old cell wall structure is destroyed, new substances would be added to ensure the integrity of the cell wall. The proportion of glucan side chains connected with β-1,6-glycosidic bonds in β-glucan was increased during the stipe rapid elongation stage in *A. bisporus*. We believe that *FfJmhy* significantly promotes the transcription of *Gs6* during elongation and increases the synthesis of GS6 in stipe cells and cell wall synthesis, thereby ensuring the integrity of the cell wall. In addition, the expansin-like protein, as a special protein in the cell wall of fungi, participates in the structural formation of the cell wall [59]. *FfJmhy* actively regulates the transcription of expansin-like genes to better promote relaxed cell wall extension and plays an auxiliary regulatory role in the process of stipe cell wall elongation.

Remodeling the cell wall is very beneficial to rapid cell elongation. *FfJmhy* can regulate the mycelial growth rate and the stipe elongation rate by improving rapid cell elongation rather than cell proliferation. On a macro level, *FfJmhy* can positively regulate the elongation rate and total length of the stipe, and does not affect the number of the fruiting body. For the industrialized cultivation of *F. filiformis* and other agaric fungi, there is a hope that the elongation rate and length of the stipe can be regulated to achieve the best commercial character and market timing, which could be achieved by regulating *FfJmhy* and its related regulatory networks.

## Data Availability

All experimental data in this study will be made available upon reasonable request from readers.

## Supplementary Materials

The following supporting information can be downloaded at: https://www.mdpi.com/article/10.3390/jof8050477/s1, Table S1: The primers used in this study; Table S2: The information of predicted cis-elements in the *FfJmhy* promoter; Table S3: The satellite repeats number in the gene promoter; Figure S1: Stability evaluation of six internal control gene candidates by geNorm software; Figure S2: Cis-regulatory element analysis of *FfJmhy* promoter.
