# Supplementary material for: The Chromatin Modifier Protein FfJMHY Plays an Important Role in Regulating the Rate of Mycelial Growth and Stipe Elongation in Flammulina filiformis
Source: J Fungi (Basel). 2022 May 3;8(5):477. doi: 10.3390/jof8050477 (PMC9147824; doi:10.3390/jof8050477)
Supplement: Supplementary file 1 [file jof-08-00477-s001.zip › jof-1625422-supplementary.pdf]

Supplementary materials:

The chromatin modifier protein FfJMHY plays an important role in regulating the rate of mycelial growth and stipe elongation in *Flammulina filiformis*

Jian Li[1,2], Yanping Shao[1,2], Yayong Yang[1,2], Chang Xu[1,2], Zhuohan Jing[1,2], Hui Li[3], Baogui Xie[2], Yongxin Tao[1,2,*]

[1] College of Horticulture, Fujian Agriculture and Forestry University, Fuzhou, 350002, Fujian, China; jianli0226@163.com (J.L.); huyx98@163.com (Y.S.); yangyy1839@163.com (Y.Y.); xc0406xn@163.com (C.X.); chenyz805236513@163.com (Z.J.)

[2] Mycological Research Center, College of Life Sciences, Fujian Agriculture and Forestry University, Fuzhou, 350002, Fujian, China; mrcfafu@163.com (B.X.)

[3] Institute of Cash Crops, Hebei Academy of Agriculture and Forestry Sciences, Shijiazhuang, 050051, Hebei, China; lihuiviphappy@163.com (H.L.)

* Correspondence: taoyongxinmuse@163.com (Y.T.) Tel.: 0086-0591-83789281

Table S1. The primers used in this study

Primer	Sequence (5′ to 3′)	Description
FfACTB-qF<br>FfACTB-qR	GATCGTATGCAGAAGGAGTTGACAC<br>CCACTCTCGTCGTACTCTTGCTTG	Internal control gene for RT-qPCR
FfGAPDH-qF<br>FfGAPDH-qR	TCAATGCGACGAGTAAAGAGAGG<br>CATAGGTCCCACATCTACATTTCG	Internal control gene for RT-qPCR
FfRas-qF<br>FfRas-qR	GTTTCCGTTGTTGACCTTGTGTGCC<br>CGAAGTTGCCGTTGAGCTGGATAC	Internal control gene for RT-qPCR
FfJmhy-qF<br>*FfJmhy*-qR	GTCGTACTTTACTGCTCTGTTCG<br>GATCTTGTTTCTCGTGGTCTGG	Detecting expression level of *FfJmhy* gene by RT-qPCR
FfJmhy-oeF<br>FfJmhy-oeR	CACAACAACTTATCactagtACTCCGGGCGCCACAACGAC<br>ATGCCAATTCTAGAgggccCCCTCTCACACAACGAACAGC	Constructing *FfJmhy* overexpression vector
FfJmhy-antisense-F<br>FfJmhy-antisense-R	TTACTGATGCaattgACCAGAAGTTACTCACCTGTCCGAA<br>ATGCCAATTCTAGAgggccCGCCTTGCTAATAATATACAG	Constructing *FfJmhy* knockdown precursor
FfJmhy-sense-F<br>FfJmhy-sense-R	CACAACAACTTATCactagtGCCTTGCTAATAATATACAG<br>CTGGTCaattgCATCAGTAAGGTG	Constructing *FfJmhy* knockdown vector
Hpt-F<br>Hpt-R	CTATTCCTTTGCCCTCGG<br>ATGAAAAAGCCTGAACTCACC	Hygromycin gene was used for transformation verification
Ffexg1-qF<br>*Ffexg1*-qR	GAACTATTCCCAAGACGCTCTC<br>GACACTCAACTGGTACGTCACTC	Detecting expression level of *Ffexg1* gene by RT-qPCR
Ffexg2-qF<br>*Ffexg2*-qR	GTCTCTACAGCTTCTTCCAAAATTACG<br>CGTTTCTGTTATTCGCTTGGTTTGC	Detecting expression level of *Ffexg2* gene by RT-qPCR
Ffexg3-qF<br>*Ffexg3*-qR	AGATGAATGTAGCCCTCGGTCTTC<br>GTTTAGGCTGTAGATGGTTATGGAG	Detecting expression level of *Ffexg3* gene by RT-qPCR
Ffgs6-qF<br>*Ffgs6*-qR	CTCCTCTTAGTGTCAGCAATAGTC<br>GGTAGTCCTGACGTGAATGTATAG	Detecting expression level of β-1, 6-glucan synthase (*Ffgs6*) gene by RT-qPCR
Ffgene5816-qF<br>*Ffgene5816*-qR	TTCGTTCGCTAATACCAAGG<br>TGACACCATTTCCTTCTTCC	Detecting expression level of *Ffgene5816* gene by RT-qPCR
Ffgene8937-qF<br>*Ffgene8937*-qR	TCTACTCCTACAAGACTCTTCC<br>GATGTACTGCGACTTGATCC	Detecting expression level of *Ffgene8937* gene by RT-qPCR
Ffgene7763-qF<br>*Ffgene7763*-qR	GGATGAACTGCTCTCGAAGATG<br>CGACATCCCATGCTGAGATATAA	Detecting expression level of *Ffgene7763* gene by RT-qPCR
Ffgene2644-qF<br>*Ffgene2644*-qR	CTCGACGATACGGACAACTATCT<br>TCCTTGCCGATCCAAGGTATAG	Detecting expression level of *Ffgene2644* gene by RT-qPCR
Ffgene9895-qF<br>*Ffgene9895*-qR	CATAGATGTCTGTGGAGTGG<br>CGAGACTATGACTTGCTTCG	Detecting expression level of *Ffgene9895* gene by RT-qPCR
Ffexpl1-qF<br>*Ffexpl1*-qR	CTCTGAGGACCTTGGTCTTATC<br>CATATGTTGAAGTTGGCTCAGG	Detecting expression level of *Ffexpl1* gene by RT-qPCR
Ffexpl2-qF<br>*Ffexpl2*-qR	GTGGTTACGATGACCTCGACAT<br>AAGGATGGAAGGACTTGCTCTC	Detecting expression level of *Ffexpl2* gene by RT-qPCR

Table S2. The information of predicted cis-elements in the *FfJmhy* promoter

Position		Matrix score	Sequence	Function	Organism
start	end				
64	68	6	AAACCA	Anaerobic response	*Zea mays*
406	411	6	CAACGG	Activating sequence	*Hordeum vulgare*
411	416	6	AACGAC	Auxin responsiveness	*Brassica oleracea*
411	417	6	AACGAC	Auxin responsiveness	*Brassica oleracea*
437	442	6	AACGAC	Auxin responsiveness	*Brassica oleracea*
523	528	6	AACGAC	Auxin responsiveness	*Brassica oleracea*
534	538	5	CAAAT	Promoter and enhancer	*Pisum sativum*
565	569	5	CAAAT	Promoter and enhancer	*Pisum sativum*
595	599	5	CAAAT	Promoter and enhancer	*Pisum sativum*
617	621	5	CAAAT	Promoter and enhancer	*Pisum sativum*
617	621	5	CGTCA	MeJA response	*Hordeum vulgare*
738	742	5	CGTCA	MeJA response	*Hordeum vulgare*
750	754	5	CGTCA	MeJA response	*Hordeum vulgare*
777	782	6	GGTTAA	Light response	*Arabidopsis thaliana*
778	783	6	CCGAAA	Low-temperature responsiveness	*Hordeum vulgare*
792	797	6	CCGAAA	Low-temperature responsiveness	*Hordeum vulgare*
826	831	6	CAACTG	Drought response	*Arabidopsis thaliana*
886	891	6	CAACTG	Drought response	*Arabidopsis thaliana*
894	898	5	CAAAT	NON-homologous region enhancer	*Pisum sativum*
898	901	4	TATA	Core promoter	*Arabidopsis thaliana*
992	995	4	TATA	Core promoter	*Arabidopsis thaliana*

Table S3. The satellite repeats in the promoters of cell wall-related enzymes encoding genes

Gene	Motif	No.of Repeats
Exg2-1	ga	4
Exg3-1	ct	4
Exg3-2	ag	5
Exg3-3	ct	4
Chi9-1	gc	4
Chi9-1	gc	4
Chi14-1	ct	4
Chi16-1	ct	4
Chi16-2	ggt	4
Expl2-1	ga	6
Expl2-2	gcc	4
Expl2-3	cca	4

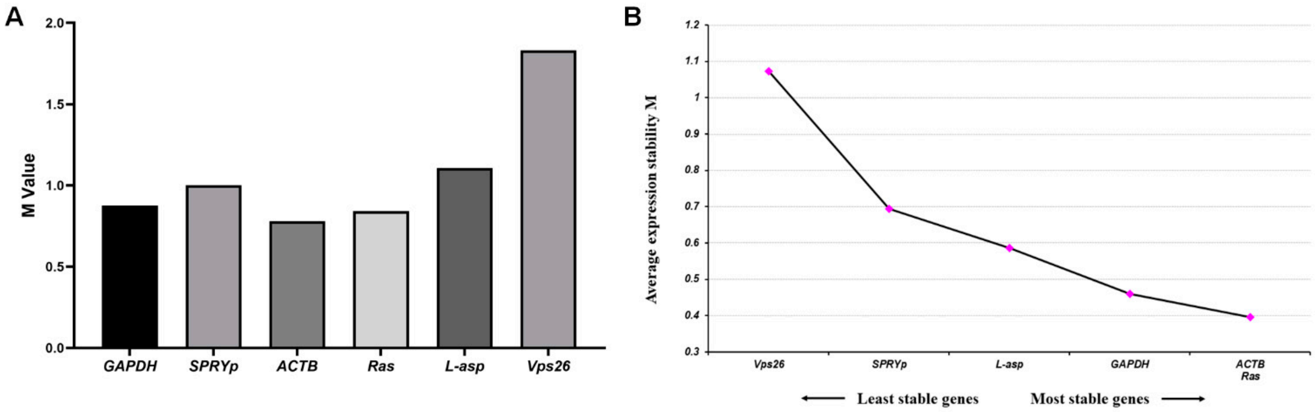

Figure S1. Stability evaluation of six internal control genes candidates by geNorm software. A) M value of six internal control genes candidates was calculated by geNorm based on Ct value of RT-qPCR. The smaller the M value, the higher the stability. B) Stability ranking of six candidate internal control genes recommended by geNorm.

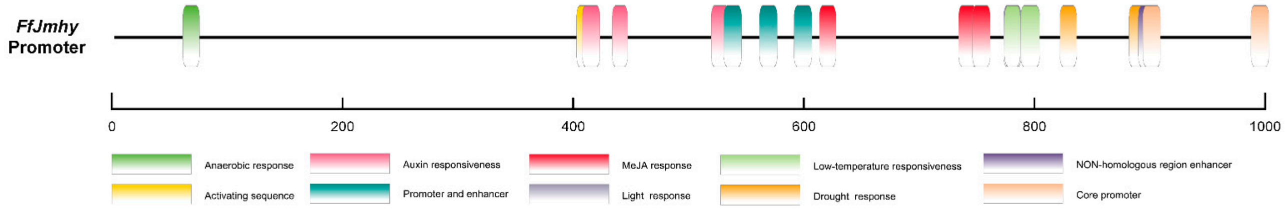

Figure S2. The predicted cis-elements in the *FfJmhy* promoter by PlantCare.
